# Facile synthesis and emission enhancement in NaLuF_4_ upconversion nano/micro-crystals via Y^3+^ doping

**DOI:** 10.1038/s41598-017-14228-9

**Published:** 2017-10-23

**Authors:** Hao Lin, Dekang Xu, Anming Li, Lu Yao, Zhiren Qiu, Shenghong Yang, Yueli Zhang

**Affiliations:** State Key Laboratory of Optoelectronic Materials and Technologies, School of Materials Science and Engineering, School of Physics, Sun Yat-sen University, Guangzhou, 510275 P. R. China

## Abstract

A series of Y^3+^-absent/doped NaLuF_4_:Yb^3+^, Tm^3+^ nano/micro-crystals were prepared via a hydrothermal process with the assistance of citric acid. Cubic nanospheres, hexagonal microdisks, and hexagonal microprisms can be achieved by simply adjusting the reaction temperature. The effect of Y^3+^ doping on the morphology and upconversion (UC) emission of the as-prepared samples were systematically investigated. Compared to their Y^3+^-free counterpart, the integrated spectral intensities in the range of 445–495 nm from α-, β-, and α/β-mixed NaLuF_4_:Yb^3+^, Tm^3+^ crystals with 40 mol% Y^3+^ doping are increased by 9.7, 4.4, and 24.3 times, respectively; red UC luminescence intensities in the range of 630–725 nm are enhanced by 4.6, 2.4, and 24.9 times, respectively. It is proposed that the increased UC emission intensity is mainly ascribed to the deformation of crystal lattice, due to the electron cloud distortion in host lattice after Y^3+^ doping. This paper provides a facile route to achieve nano/micro-structures with intense UC luminescence, which may have potential applications in optoelectronic devices.

## Introduction

Optical upconversion (UC) is an anti-Stokes process that two or more low-energy photons can be converted into a single high-energy photon^1^. Rare-earth (RE) doped UC materials show many advantages, including high photochemical stability, low toxicity and long luminescence lifetimes^2–6^, which may have great potential applications in fields such as biological imaging, multi-dimensional displays, optical temperature sensors and solar cells^7–10^. However, compared to downconversion materials, the main shortcoming of UC materials is their low luminescence efficiency. Thus, an effective strategy to enhance the UC luminescence intensity is urgently needed. In recent years, many kinds of methods have been used to achieve efficient UC luminescence. For instance, Zhao *et al*. reported the enhanced red UC emission in Mn^2+^ doped NaYF_4_: Yb/Er nanoparticles, due to the efficient energy transfer between Er^3+^ and Mn^2+^ ^11^. Tan *et al*. demonstrated NaYbF_4_:Tm^3+^ and NaYbF_4_:Er^3+^ nanocrystals with the enhanced red UC luminescence, which is attributed to the cross relaxation effect among the activators at high activator content^12^. As is known, the UC emission of RE doped materials is remarkably affected by the crystal field symmetry around activators^13^, and the asymmetric environment of activators can result in the emission enhancement. For instance, Zhao’s group reported Li^+^ doped GdF_3_:Yb^3+^, Er^3+^ nanocrystals with the enhanced red UC luminescence, which was caused by the decrease of local crystal field symmetry around activators after Li^+^ doping^14^. Rai *et al*. demonstrated the enhanced green UC emission in Li^+^ doped Y_2_O_3_:Yb^3+^/Er^3+^ nanocrystals^15^. Yin *et al*. reported Mo^3+^ doped NaYF_4_: Yb/Er nanocrystals with 6 and 8 times enhancement of green and red UC emissions, due to the lattice distortion after Mo^3+^ doping^16^. In order to obtain efficient UC emission, the selection of excellent host material is essential. With the similar crystalline plane, NaYF_4_ and NaLuF_4_ have been considered as the outstanding host matrix for UC processes, due to their high thermal stability, low phonon energy and high refractive index^17–21^. As is known, the ionic radius of Y^3+^ (0.89 Å) is larger than that of Lu^3+^ (0.85 Å), thus Y^3+^ doping may cause the expansion of NaLuF_4_ host lattice, leading to the distortion of local symmetry around activators. Consequently, Y^3+^ doping is an effective approach for enhancing the UC emission intensity in NaLuF_4_-based system. In addition, due to the small difference in ionic radius between Y^3+^ and Lu^3+^, the phase transformation does not occur during introducing Y^3+^ in NaLuF_4_ crystals, which would be favorable to maintain the stability of crystal structure. However, there is no report on the increase of UC luminescence intensity in NaLuF_4_-based system via Y^3+^ doping.

In this paper, in order to obtain different structures of NaLuF_4_ nano/micro-crystals before Y^3+^ doping, the influence of reaction temperature on the phase of Y^3+^-absent NaLuF_4_ crystals is studied. It is found that cubic nanospheres, hexagonal microdisks and hexagonal microprisms can be achieved with the higher temperature. α-, β-, and α/β-mixed NaLuF_4_:Yb^3+^, Tm^3+^ crystals with Y^3+^ doping show the significant enhancement of UC emissions relative to Y^3+^-absent samples under 980 nm excitation at room temperature. The proposed mechanisms of UC emission enhancement and shape evolution through introducing Y^3+^ are presented.

## Results and Discussion

### Phase and morphology

**Figure 1:**
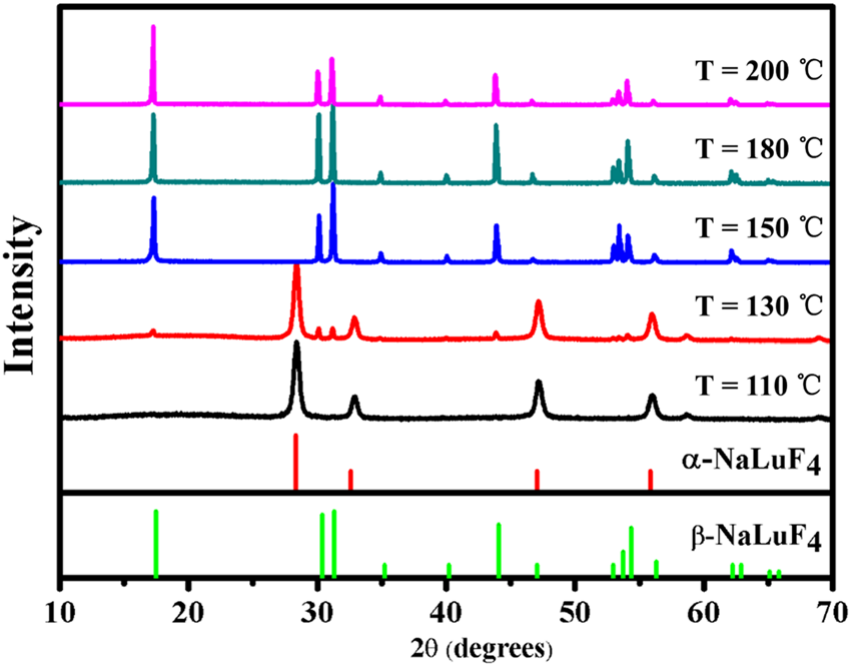
XRD patterns of Y^3+^-absent NaLuF_4_:Yb^3+^, Tm^3+^ nano/micro-crystals prepared at different reaction temperatures (110 °C, 130 °C, 150 °C, 180 °C, and 200 °C) for 12 h. The vertical red and green lines are the standard profiles of α-NaLuF_4_ (JCPDS 27-0725) and β-NaLuF_4_ (JCPDS 27-0726), respectively.

**Figure 2:**
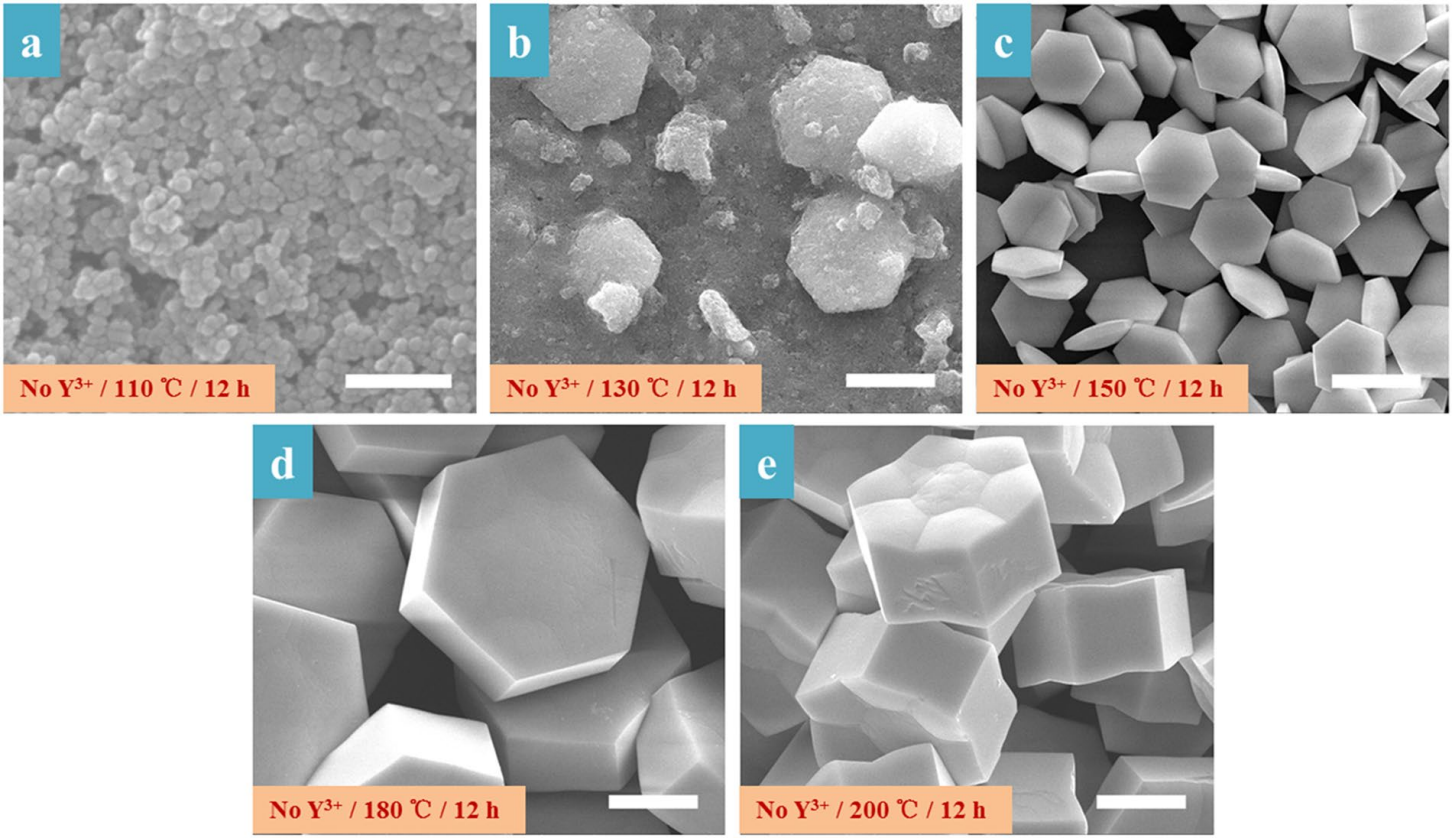
SEM images of Y^3+^-absent NaLuF_4_:Yb^3+^, Tm^3+^ nano/micro-crystals prepared at different reaction temperatures for 12 h. (**a–e**) Refer to 110 °C, 130 °C, 150 °C, 180 °C, and 200 °C, respectively. Scale bars are 200 nm for (**a**), and 5 μm for (**b–e**).

First, in order to obtain diverse structures of NaLuF_4_ nano/micro-crystals before Y^3+^ doping, the influence of reaction temperature on the crystal structure of Y^3+^-absent NaLuF_4_ crystals is studied. The XRD patterns and the corresponding SEM images of Y^3+^-absent NaLuF_4_:Yb^3+^, Tm^3+^ nano/micro-crystals prepared at different reaction temperatures for 12 h are displayed in Figs 1 and 2, respectively. As can be seen from Fig. 1, pure α-NaLuF_4_ (JCPDS 27-0725) is formed at 110 °C. The related SEM image (Fig. 2a) shows that the sample is composed of a large number of small cubic nanospheres with an average diameter of 17 nm. At higher reaction temperature of 130 °C, α/β-mixed NaLuF_4_ appears in the XRD pattern, indicating that the crystals partially change from α to β phase. Correspondingly, the SEM image of Fig. 2b exhibits two obvious particle morphologies containing small α-NaLuF_4_ nanospheres and large β-NaLuF_4_ microdisks with a mean diameter of 7.63 μm. After being treated at 150 °C, the corresponding XRD result demonstrates that pure β-NaLuF_4_ (JCPDS 27-0726) can be obtained. The corresponding sample is composed of a large amount of hexagonal microdisks with regularity and smooth surfaces, and the small cubic nanoparticles completely disappear, as presented in Fig. 2c. The average length and diameter of the disks are 0.51 μm and 4.80 μm, respectively. When the reaction temperature further increases to 180 °C and 200 °C, there still only exists hexagonal phase in the XRD patterns. The corresponding SEM images (Fig. 2d and e) show the homogeneous short hexagonal microprisms with an average size of 4.36 μm and 6.06 μm in length; 12.46 μm and 10.51 μm in diameter, respectively. The ratios of length to diameter (*L/D* ratios) are calculated to be about 0.11 (150 °C), 0.35 (180 °C), and 0.58 (200 °C). From the above analysis, it can be concluded that higher reaction temperature favors the formation of NaLuF_4_ crystals with hexagonal phase, which is ascribed to the fact that higher temperature favors the nucleation and the crystal growth^25^. The *L/D* ratio of β-NaLuF_4_ microcrystals is enhanced as the temperature increases from 150 °C to 200 °C. As is known, β-NaLuF_4_ has a high anisotropic structure^26^. The growth rate along [10ī0] direction is lower than that along [0001] direction at higher temperature due to Cit^3−^ absorbs onto the {$10\bar{{\bf{1}}}0$} facets more strongly than the {0001} facets, thus results in the increase of *L/D* ratio and the shape evolution from disks to prisms.

In order to reveal the effect of Y^3+^ doping on the morphology and UC emission of NaLuF_4_ crystals, a series of Y^3+^ doped α-, β- and α/β-mixed NaLuF_4_:Yb^3+^, Tm^3+^ nano/micro-crystals were synthesized.

**Figure 3:**
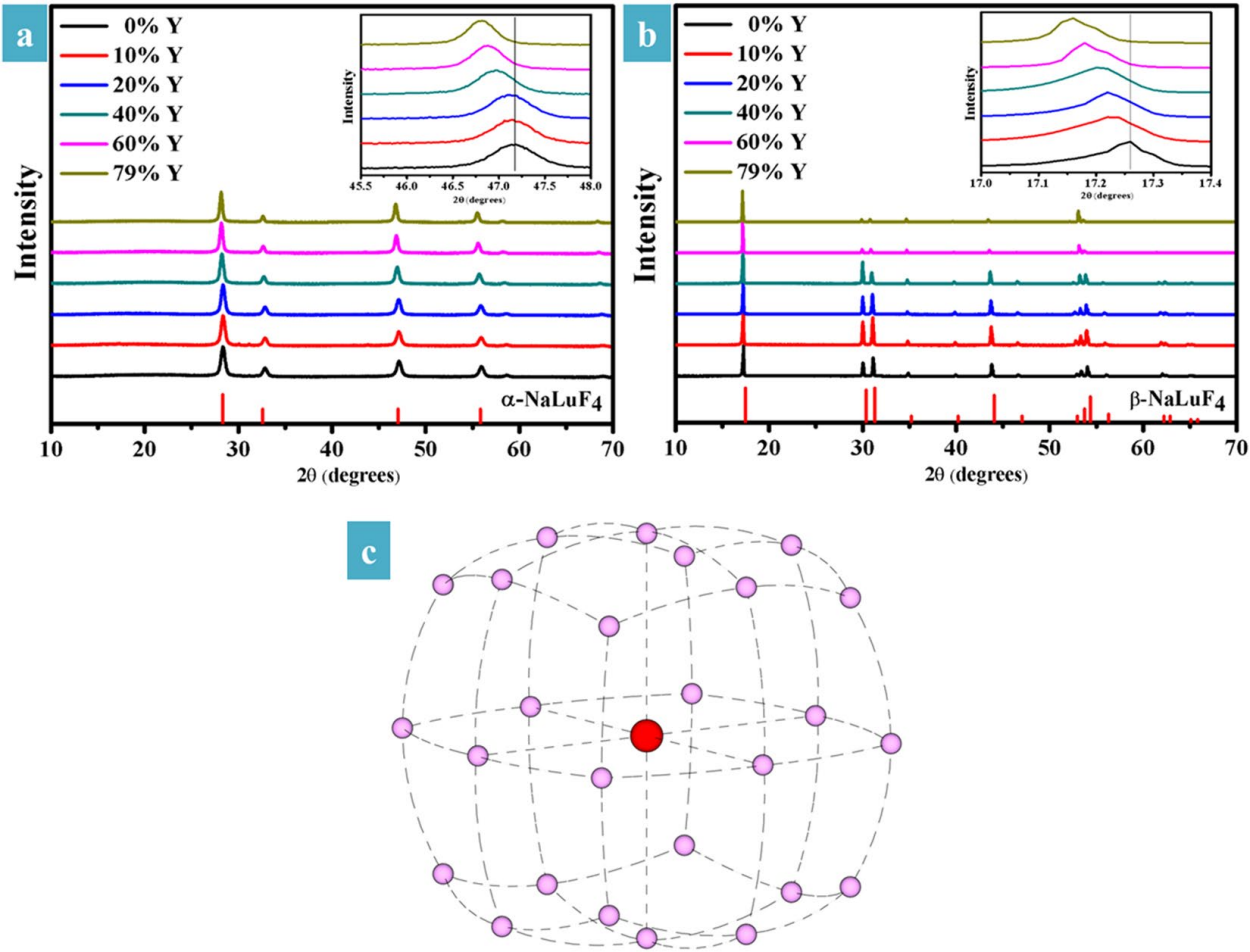
XRD patterns (**a** and **b**) of different Y^3+^ doped α-NaLuF_4_:Yb^3+^, Tm^3+^ nanocrystals and β-NaLuF_4_:Yb^3+^, Tm^3+^ microcrystals prepared at 110 °C and 200 °C for 12 h; and possible change in the NaLuF4 crystal lattice after Y^3+^ doping (**c**). The insets of (**a** and **b**) are their main diffraction peaks. The vertical red lines are the standard profiles of α-NaLuF_4_ (JCPDS 27-0725) and β-NaLuF_4_ (JCPDS 27-0726), respectively.

Figure 3(a and b) show the XRD patterns of α-NaLuF_4_:Yb^3+^, Tm^3+^ nanocrystals and β-NaLuF_4_:Yb^3+^, Tm^3+^ microcrystals introduced with different Y^3+^ contents prepared at 110 °C and 200 °C for 12 h, respectively. As can be seen, pure cubic phase (Fig. 3a) and pure hexagonal phase (Fig. 3b) can be obtained even Y^3+^ content increases up to 79 mol% (the Y^3+^-free samples have been shown in Fig. 1). No extra peaks can be observed, which indicates that Y^3+^ doping has no influence on the crystal structure of cubic-phase nanocrystals and hexagonal-phase microcrystals. As demonstrated in the insets of Fig. 3(a and b), with the Y^3+^ content increases from 0 to 79 mol%, the main diffraction peaks of α and β phases move to lower angles. According to Bragg’s law 2*d* sin*θ* = *nλ*, where *d* represents the interplanar distance, *θ* represents the diffraction angle, and *λ* represents the diffraction wavelength. When Y^3+^ doped into the lattice, Lu^3+^ can be substituted by the relatively large Y^3+^, resulting in the expansion of NaLuF_4_ host lattice (Fig. 3c), thus the interplanar distance increases and diffraction angle decreases. The values of the lattice constants and unit-cell volumes of α-NaLuF_4_:20%Yb^3+^, 1%Tm^3+^ doped with different concentrations of Y^3+^ calculated according to XRD results are shown in Table 1, the higher unit-cell volumes are caused by the larger ionic radius of Y^3+^ substituting Lu^3+^. Importantly, the lattice expansion may cause the distortion of local symmetry around Tm^3+^, which would break the forbidden transition of Tm^3+^, and consequently enhancing the UC emission intensity^27^. The above XRD results are well consistent with the corresponding SEM images.

**Table 1:** The lattice constants and unit-cell volumes of α-NaLuF_4_:20%Yb^3+^, 1%Tm^3+^ doped with different concentrations of Y^3+^.

	0% Y^3+^	10% Y^3+^	20% Y^3+^	40% Y^3+^	60% Y^3+^	79% Y^3+^
a/Å	5.4461	5.4496	5.4515	5.4663	5.4796	5.4852
unit-cell volume (Å^3^)	161.53	161.84	162.02	163.33	164.53	165.03

**Figure 4:**
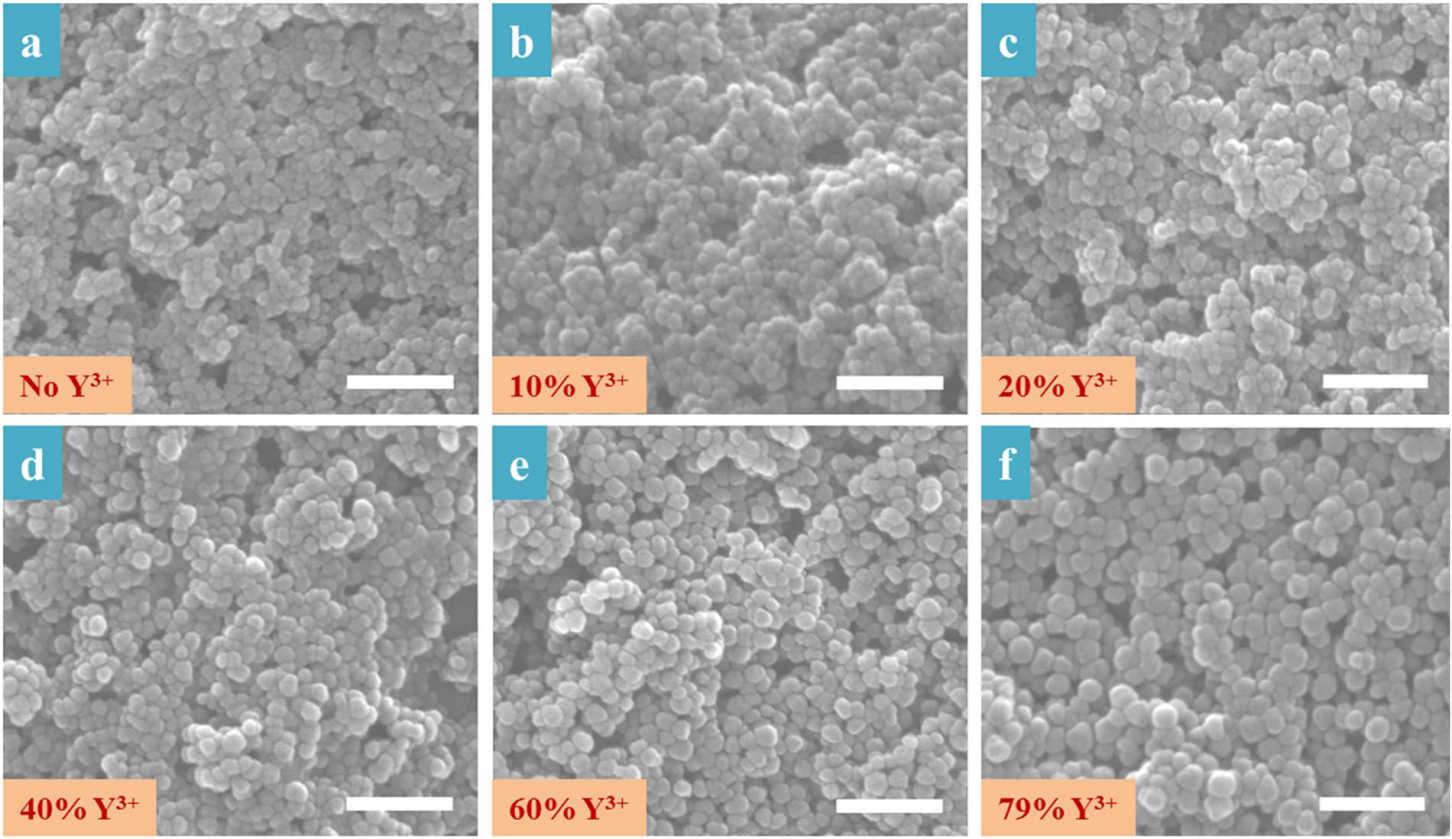
SEM images of different Y^3+^ doped α-NaLuF_4_:Yb^3+^, Tm^3+^ nanocrystals prepared at 110 °C for 12 h. (**a**–**f**) Refer to 0, 10, 20, 40, 60, and 79 mol%, respectively. Scale bars = 200 nm.

**Figure 5:**
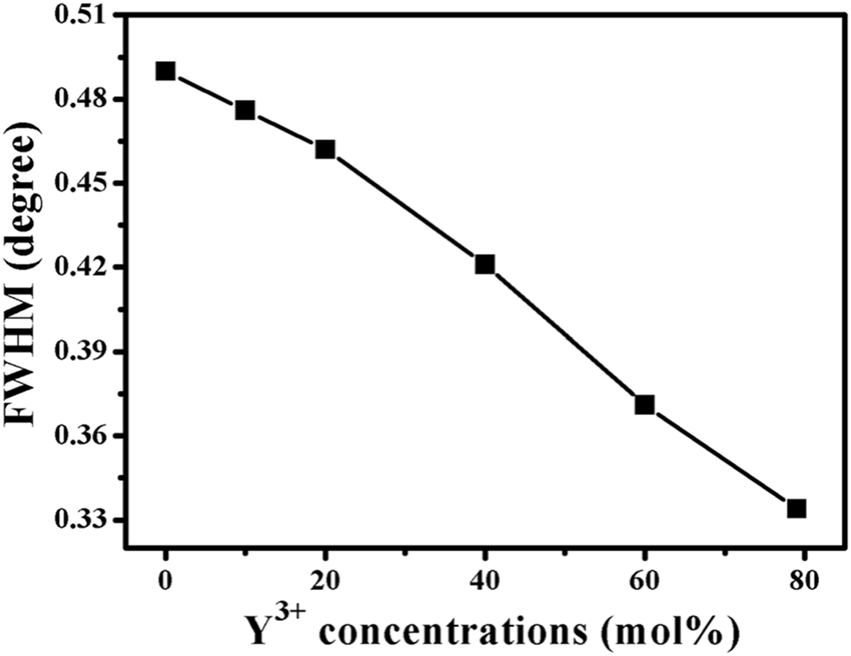
FWHM of 28.16° peak *vs*. concentrations of Y^3+^ in Y^3+^ doped α-NaLuF_4_:Yb^3+^, Tm^3+^ nanocrystals.

As shown in Fig. 4(a–f), the Y^3+^ doped α-NaLuF_4_ nanoparticles are composed of a great deal of small cubic nanospheres (the Y^3+^- absent sample has been shown in Fig. 2a). The full width at half maximum (FWHM) was gradually narrowed with the Y^3+^ concentration increases up to 79 mol%, as presented in Fig. 5. The average crystalline sizes can be calculated based on Scherrer’s equation: *D* = 0.89*λ*/(*β*cos*θ*), where *D* is the crystallite size, *λ* represents the wavelength of the X-ray, *β* stands for the corrected half width of the diffraction peak, and *θ* is the diffraction angle. The factor 0.89 is the characteristic of a spherical particle. Thus, the mean diameters (Table 2) of the spheres were calculated to be about 17 nm, 17 nm, 18 nm, 19 nm, 22 nm, and 24 nm, respectively. From the above results, it can be seen that the replacement of Lu^3+^ by larger Y^3+^ may lead to the increasing size of cubic-phase nanospheres.

**Table 2:** The mean size of different Y^3+^ doped α-NaLuF_4_:Yb^3+^, Tm^3+^ nanospheres prepared at 110 °C for 12 h.

	0% Y^3+^	10% Y^3+^	20% Y^3+^	40% Y^3+^	60% Y^3+^	79% Y^3+^
size (nm)	17	17	18	19	22	24

**Figure 6:**
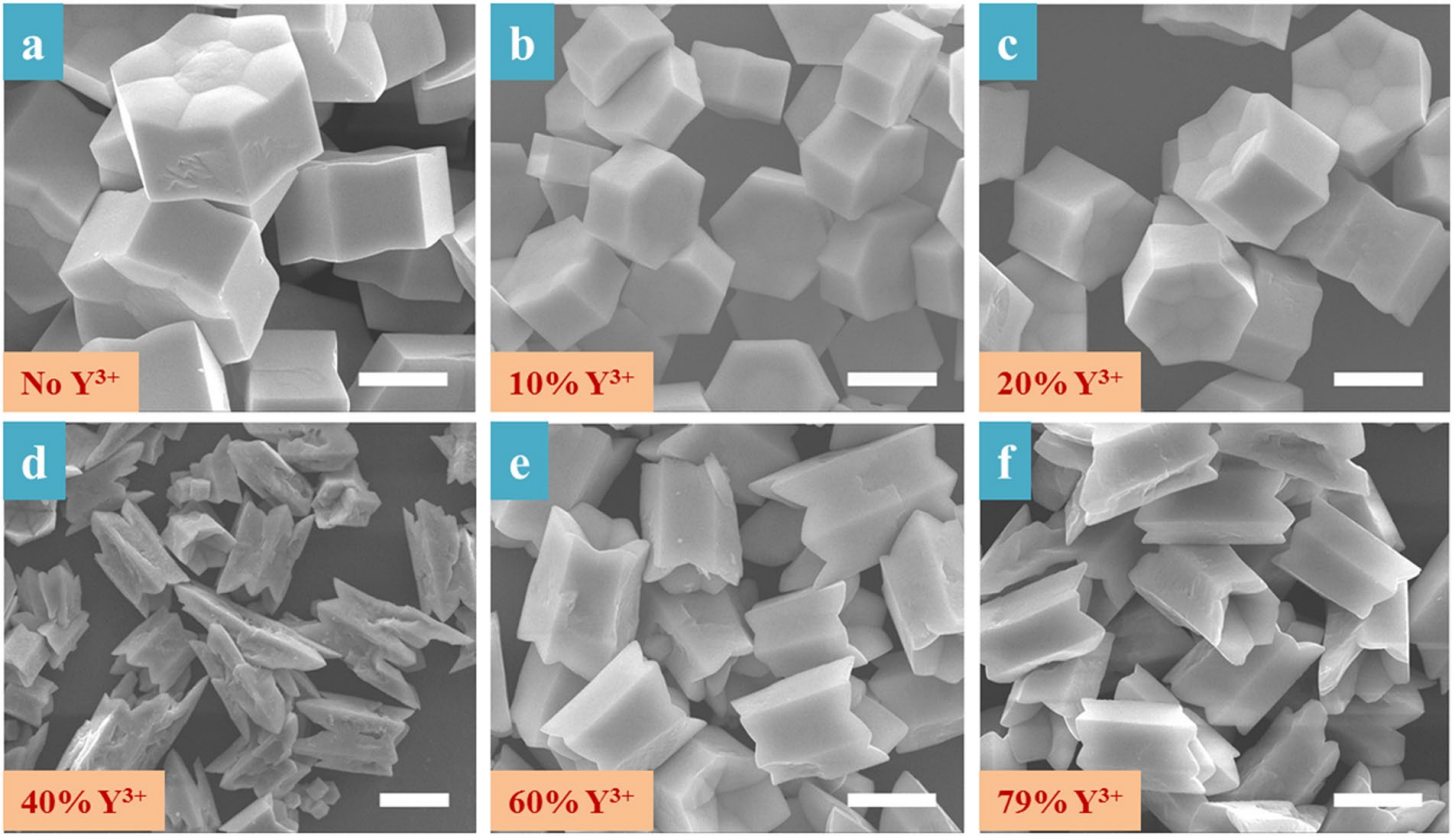
SEM images of different Y^3+^ doped β-NaLuF_4_:Yb^3+^, Tm^3+^ microcrystals prepared at 200 °C for 12 h. (**a–f**) Refer to 0, 10, 20, 40, 60, and 79 mol%, respectively. Scale bars are 5 μm for (**a–c, e** and **f**), and 10 μm for (**d**).

The SEM images of Y^3+^ doped β-NaLuF_4_ microparticles are displayed in Fig. 6(a–f). As exhibited in Fig. 6a, the Y^3+^-free sample has been shown in Fig. 2e. As the Y^3+^ concentration increases from 10 to 20 mol%, short hexagonal microprisms with regularity and uniformity are obtained, as presented in Fig. 6(b and c). On average, the prisms have a length of 3.01 μm and 4.81 μm; a diameter of 6.72 μm and 7.42 μm, respectively. When the Y^3+^ concentration increases to 40 mol%, irregular hexagonal microprisms with coarse surfaces are shown in Fig. 6d. The average length of the prisms is 14.08 μm, and the average diameter is 11.02 μm. With the Y^3+^ content further increases to 60 and 79 mol% [Fig. 6(e and f)], the corresponding samples consist of hexagonal microprisms with scrappy ends and concave centers on the top/bottom surfaces. The prisms have a mean size of 7.78 μm and 7.71 μm in length; 5.98 μm and 5.10 μm in diameter, respectively. The *L*/*D* ratios are calculated to be about 0.45, 0.65, 1.28, 1.30, and 1.51 when the Y^3+^ content is 10, 20, 40, 60, and 79 mol%. Thus, the *L/D* ratio of hexagonal microprisms is increased as the Y^3+^ content increases from 10 to 79 mol%. Under our experimental condition, the chelated Lu^3+^-Cit^3−^ complex and Y^3+^-Cit^3−^ complex were formed. As is known, both β-NaLuF_4_ and β-NaYF_4_ have high anisotropic structures. From Fig. 6a (Lu^3+^ = 79 mol%, Y^3+^ = 0 mol%) and Fig. 6f (Lu^3+^ = 0 mol%, Y^3+^ = 79 mol%), it can be clearly seen that the *L/D* ratio of β-NaYF_4_ is larger than that of β-NaLuF_4_. Thus, the *v*_1_ */v*_2_ ratio of β-NaYF_4_ is higher than that of β-NaLuF_4_ under the same experimental conditions (*v*_1_ is the growth rate along [0001] direction, *v*_2_ is the growth rate along [$10\bar{{\bf{1}}}0$] direction), leading to the enhancement of *L/D* ratio and the morphology evolution from short hexagonal microprisms to long hexagonal microprisms when the Y^3+^ concentration increases from 10 to 79 mol%. According to Liu *et al*.’s report about the density functional theory calculation on Gd^3+^ doped NaYF_4_:Yb^3+^, Er^3+^ nanoparticles, the electron charge density in host lattice changes after Y^3+^ is substituted by Gd^3+^ in the crystal lattice^28^. Under our synthesis conditions, the replacement of Lu^3+^ by larger Y^3+^ is similar to the substitution of Y^3+^ by larger Gd^3+^. Thus, it is creditable that Y^3+^ doped into NaLuF_4_ host lattice may change the electron charge density, leading to the electron cloud distortion in crystal lattice, which would cause the deformation of crystal lattice. The change in crystal lattice may result in the formation of irregular and distorted hexagonal microprisms with coarse surfaces when the Y^3+^ content is 40 mol%.

**Figure 7:**
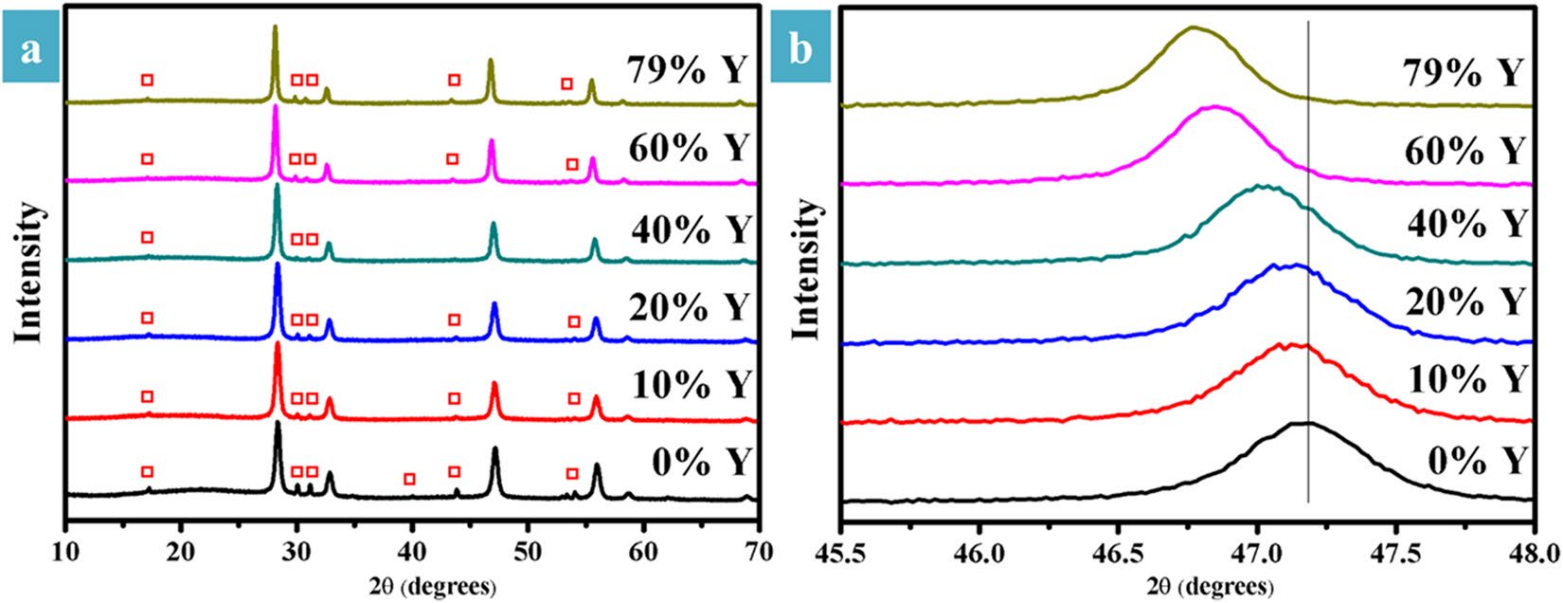
XRD patterns (**a**) and the main diffraction peak (**b**) of different Y^3+^ doped α/β-mixed NaLuF_4_:Yb^3+^, Tm^3+^ nano/micro-crystals prepared at 130 °C for 12 h. (stands for the peaks of β phase).

**Figure 8:**
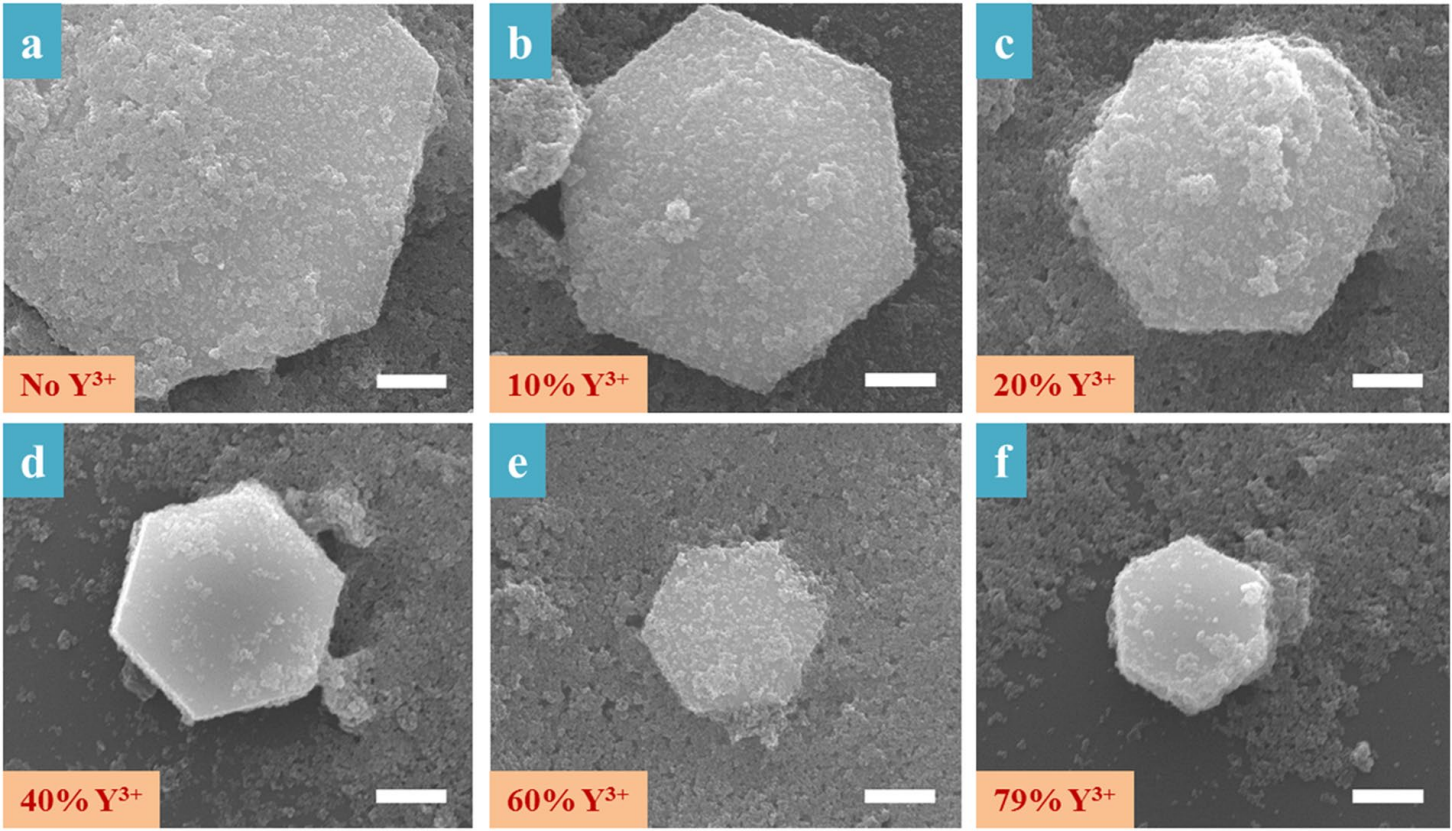
SEM images (**a**–**f**) of different Y^3+^ doped α/β-mixed NaLuF_4_:Yb^3+^, Tm^3+^ nano/micro-crystals prepared at 130 °C for 12 h. Scale bars = 1 μm.

Figure 7 shows the XRD patterns (a) and the main diffraction peak (b) of different Y^3+^ doped α/β-mixed NaLuF_4_:Yb^3+^, Tm^3+^ nano/micro-crystals prepared at 130 °C for 12 h. As shown in Fig. 7a, all samples are composed of a mixture of cubic and hexagonal phases (the Y^3+^-free sample has been shown in Fig. 1). Figure 7b displays the main diffraction peak of cubic phase shifts towards lower angles as the Y^3+^ content increases from 0 to 79 mol%, which is mainly attributed to the expansion of crystal lattice after Lu^3+^ is replaced by the relatively large Y^3+^. The shifting peak reveals that Y^3+^ can be doped into the host lattice. The corresponding SEM images [Fig. 8(a–f)] present two distinct particle morphologies including large microdisks (hexagonal phase) and small nanoparticles (cubic phase). It can be obviously seen that numerous spherical nanoparticles are attached on the surfaces of microdisks. The corresponding diameters of the disks are 7.63 μm, 5.64 μm, 4.79 μm, 3.50 μm, 2.66 μm, and 2.33 μm, respectively. The reduced diameter of the disks can be ascribed to the fact that β-NaYF_4_ has higher *v*_1_ */v*_2_ ratio than β-NaLuF_4_ under the same experimental conditions.

**Figure 9:**
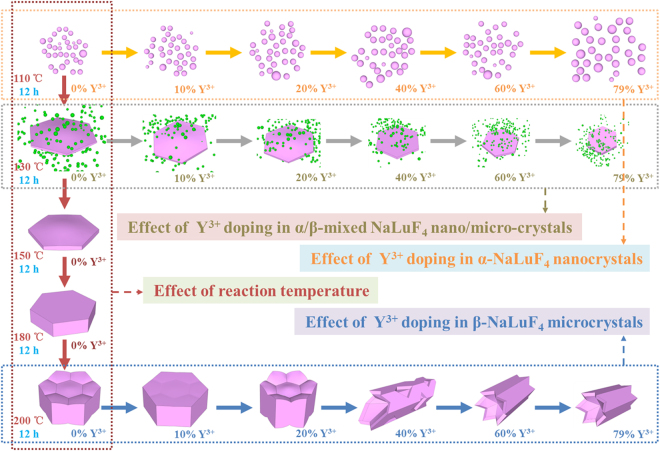
Schematic illustration for the formation processes of Y^3+^-absent/doped NaLuF_4_:Yb^3+^, Tm^3+^ nano/micro-crystals synthesized under different experimental conditions.

The above results demonstrate that reaction temperature has a significant effect on the crystal structure of the products, and Y^3+^ doping may cause the size-tuning and shape evolution of the crystals. Figure 9 summarizes the formation processes of Y^3+^-absent/doped NaLuF_4_:Yb^3+^, Tm^3+^ nano/micro-crystals synthesized under different experimental conditions.

### UC photoluminescence properties

**Figure 10:**
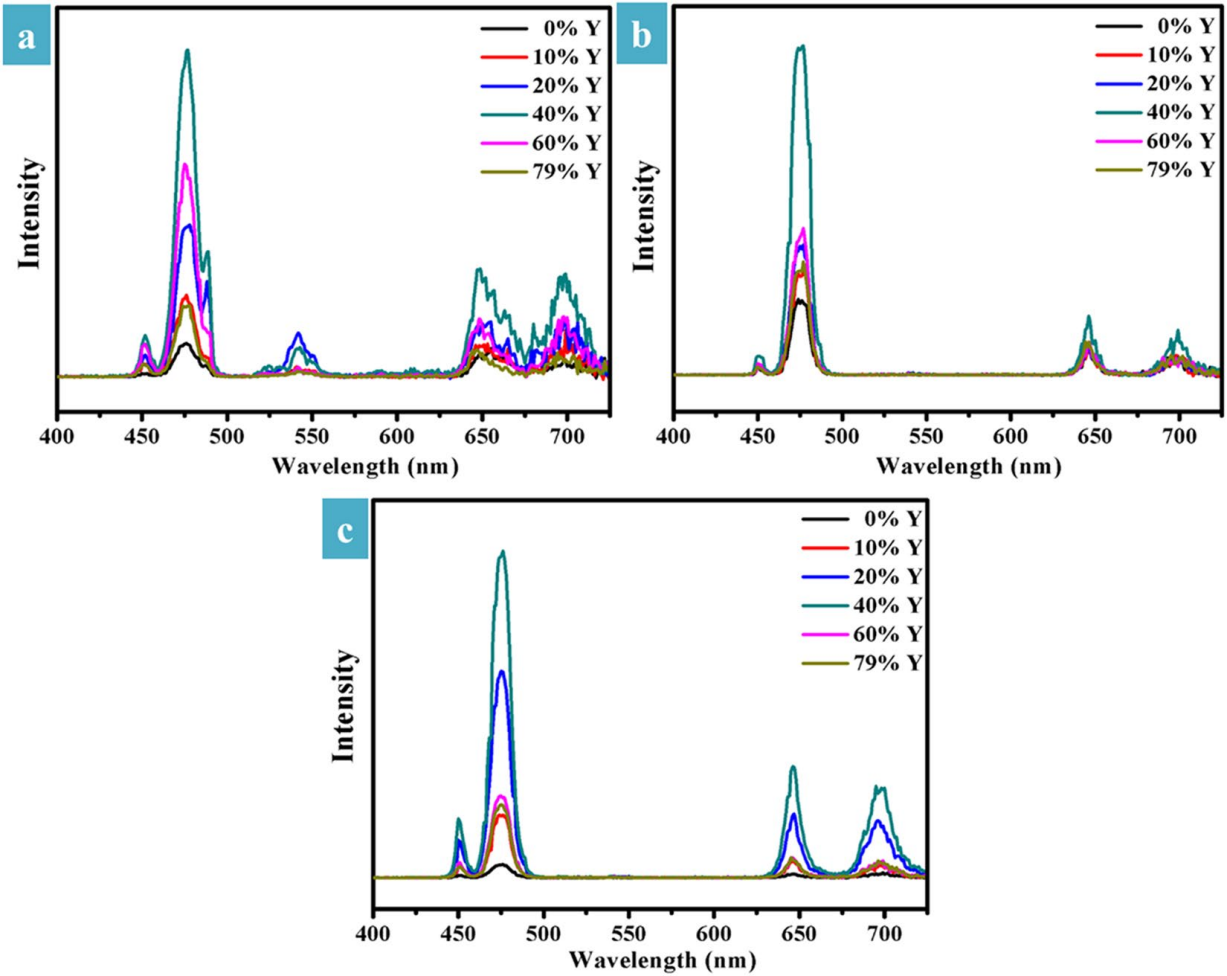
UC luminescence spectra (under 980 nm excitation) of different Y^3+^ doped α-NaLuF_4_:20%Yb^3+^, 1%Tm^3+^ nanocrystals (**a**), β-NaLuF_4_:20%Yb^3+^, 1%Tm^3+^ microcrystals (**b**), and α/β-mixed NaLuF_4_:20%Yb^3+^, 1%Tm^3+^ nano/micro-crystals (**c**) prepared at 110 °C, 200 °C, and 130 °C for 12 h, respectively.

**Figure 11:**
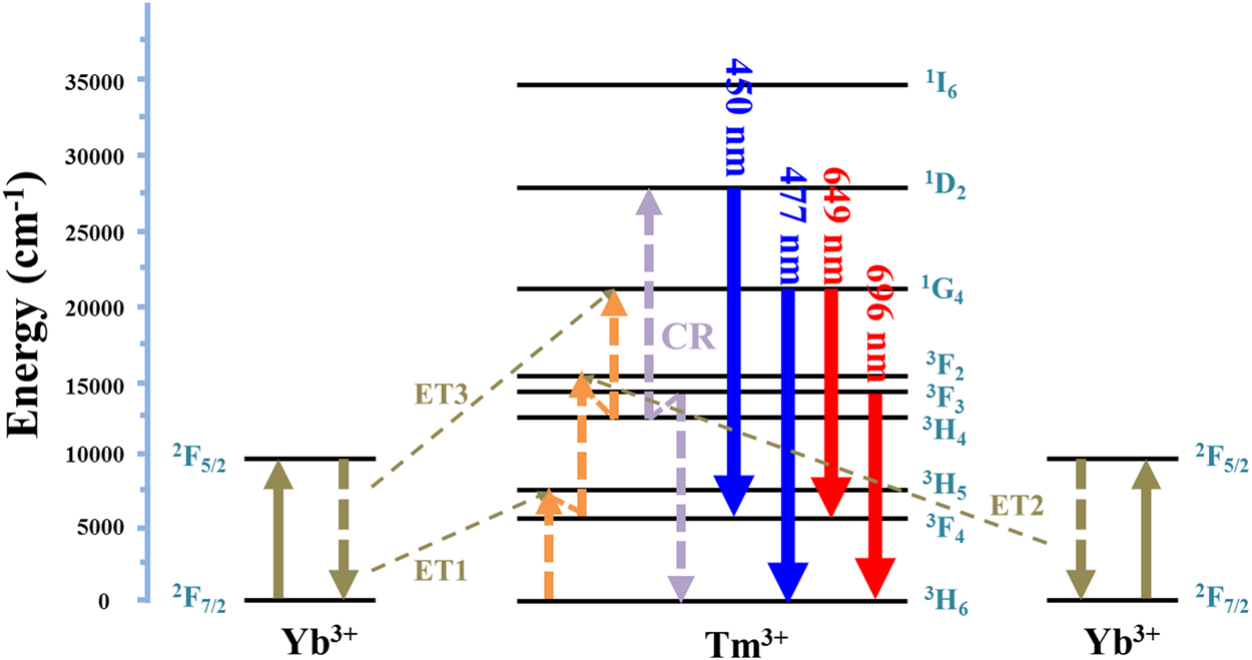
Energy level diagram showing the UC mechanisms for blue and red emissions between Yb^3+^ and Tm^3+^ under 980 nm excitation. CR and ET are the abbreviation of cross relaxation and energy transfer, respectively.

Figure 10(a–c) show the UC luminescence spectra (under 980 nm excitation at room temperature) of different Y^3+^ doped α-, β- and α/β-mixed NaLuF_4_:20%Yb^3+^, 1%Tm^3+^ nano/micro-crystals prepared at 110 °C, 200 °C and 130 °C for 12 h, respectively. Blue emissions centered at 450 nm and 477 nm are generated from the ^1^D_2_ → ^3^F_4_ and ^1^G_4_ → ^3^H_6_ transitions of Tm^3+^, respectively. Red emissions at approximately 649 nm and 696 nm correspond to the ^1^G_4_ → ^3^F_4_ and ^3^F_3_ → ^3^H_6_ transitions of Tm^3+^, respectively. The energy-level diagram of UC mechanisms for blue and red emissions between Yb^3+^ and Tm^3+^ is presented in Fig. 11. For 450 nm emission, the Tm^3+1^D_2_ level is populated by the ET1+ET2+CR processes (ET = energy transfer, CR = cross relaxation). For 477 nm and 649 nm emissions, the Tm^3+ 1^G_4_ level is populated by the ET1+ET2+ET3 processes. For 696 nm emission, the Tm^3+3^F_3_ level is populated by the ET1+ET2 processes. As can be seen from Fig. 10(a–c), the blue and red UC emission intensities are distinctly enhanced as the Y^3+^ content increases from 0 to 40 mol%, and then declined at the content of 40–79 mol%. Thus, the strongest UC luminescence intensities are observed in the samples with 40 mol% Y^3+^ doping. Compared to their Y^3+^-free samples, the integrated spectral intensities in the range of 445–495 nm from α-, β-, and α/β-mixed NaLuF_4_:20%Yb^3+^, 1%Tm^3+^ crystals with 40 mol% Y^3+^ doping are increased by 9.7, 4.4, and 24.3 times, respectively; red UC luminescence intensities in the range of 630–725 nm are enhanced by 4.6, 2.4, and 24.9 times, respectively. Under our experimental condition, the substitution of Lu^3+^ by the relatively large Y^3+^ distorts the electron charge density in host lattice, causing the lattice expansion. The deformation of crystal lattice may decrease the symmetry of the local crystal field around Tm^3+^, breaking the forbidden transition of Tm^3+^, finally favors the fast energy transfer from Yb^3+^ to Tm^3+^ ^29^. Thus, the asymmetric surrounding environment around Tm^3+^ may result in the sharp increase of UC emission intensity. Y^3+^ doping only changes the lattice constants, and the phase transformation does not occur during introducing Y^3+^ in NaLuF_4_ host lattice, due to the small difference in ionic radius between Y^3+^ and Lu^3+^. When the Y^3+^ concentration is 0 mol% (Lu^3+^ = 79 mol%) and 79 mol% (Lu^3+^ = 0 mol%), pure NaLuF_4_:20%Yb^3+^, 1%Tm^3+^ nano/micro-crystals and pure NaYF_4_:20%Yb^3+^, 1%Tm^3+^ nano/micro-crystals are formed, respectively. Consequently, the samples doped with 0 mol% Y^3+^ (Lu^3+^ = 79 mol%) and 79 mol% Y^3+^ (Lu^3+^ = 0 mol%) have the highest crystal field symmetry around Tm^3+^, and the samples doped with 40 mol% Y^3+^ (Lu^3+^ = 39 mol%) have the lowest crystal field symmetry around Tm^3+^. Due to the most asymmetric environment of Tm^3+^, α-, β- and α/β-mixed NaLuF_4_:20%Yb^3+^, 1%Tm^3+^ nano/micro-crystals with 40 mol% Y^3+^ doping have the maximum UC luminescence intensity. This phenomenon is similar to Kong *et al*.’s report about the enhanced UC emissions in Li^+^ doped NaYF_4_:Yb^3+^, Tm^3+^ nanoparticles^30^. According to the results of the experiments performed by Kong *et al*.^30^, when the Li^+^ content is below 7 mol%, Li^+^ substitutes Na^+^, causing the shrinking of host lattice; however, as the Li^+^ content increases from 7 to 15 mol%, Li^+^ begins to occupy interstitial site, leading to the expansion of crystal lattice; thus the sample with 7 mol% Li^+^ doping has the highest UC emission intensity, owing to the lowest crystal field symmetry around activators. Besides, Y^3+^ doping causes the electron cloud distortion in host lattice, resulting in the tunable size of the as-prepared samples. As is known, as for larger-size crystals, the nonradiative energy transfer processes of Tm^3+^ would decrease due to their fewer surface quenching sites^28^, which is in favor of UC emission. Thus, as for Y^3+^ doped β-NaLuF_4_:20%Yb^3+^, 1%Tm^3+^ microcrystals, the larger-size (relative to Y^3+^-absent samples) of the samples with 40 mol% Y^3+^ doping may have a small contribution to the enhancement of UC luminescence intensity.

**Figure 12:**
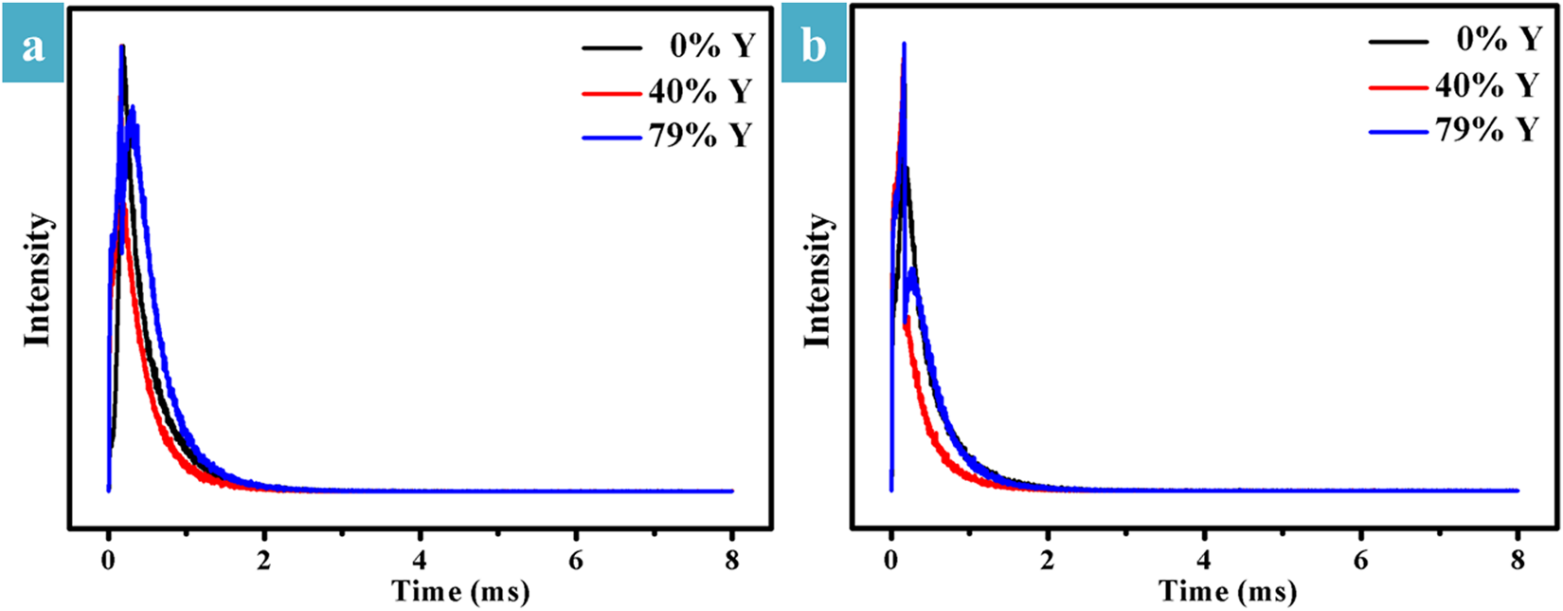
Decay curves of (**a**) ^1^G_4_ →^3^H_6_ (477 nm) and (**b**) ^1^G_4_ →^3^F_4_ (649 nm) transitions of Tm^3+^ in α-NaLuF_4_:20%Yb^3+^, 1%Tm^3+^ nanocrystals doped with 0, 40 and 79 mol% Y^3+^.

Figure 12 presents the decay curves of (a) ^1^G_4_ → ^3^H_6_ and (b) ^1^G_4_ → ^3^F_4_ transitions of Tm^3+^ in α-NaLuF_4_:20%Yb^3+^, 1%Tm^3+^ nanocrystals doped with 0, 40 and 79 mol% Y^3+^. Based on the function: *τ* = *∫I(t)dt*/*I*_*max*_, where I(t) represents the emission intensity at time t, and I_max_ represents the peak intensity in the decay curve. The calculation results (Table 3) show that τ_1_ (0, 40 and 79 mol%/477 nm) = 0.391, 0.330 and 0.541 ms. τ_2_ (0, 40 and 79 mol%/649 nm) = 0.354, 0.250 and 0.353 ms. As can be seen, the sample with 40 mol% Y^3+^ doping has the lowest luminescence lifetime of ^1^G_4_ state of Tm^3+^. It is well known that the inverse of lifetime (1/*τ*) is equal to the sum (A_r+nr_ = A_r_ + A_nr_) of radiative (A_r_) and nonradiative (A_nr_) transition probability. Thus, the lowest luminescence lifetime in the sample with 40 mol% Y^3+^ doping is mainly caused by the maximum emission intensity.

**Table 3:** Lifetimes of ^1^G_4_ → ^3^H_6_ and ^1^G_4_ → ^3^F_4_ transitions of Tm^3+^ in α-NaLuF_4_:20%Yb^3+^, 1%Tm^3+^ nanocrystals doped with 0, 40 and 79 mol% Y^3+^.

Y^3+^ content (mol%)	τ_1_/ms (^1^G_4_ → ^3^H_6_, Tm^3+^)	τ_2_/ms (^1^G_4_ → ^3^F_4_, Tm^3+^)
0	0.391	0.354
40	0.330	0.250
79	0.541	0.353

## Conclusion

In summary, cubic nanospheres, hexagonal microdisks, and hexagonal microprisms can be achieved by simply adjusting the reaction temperature. It is found that higher temperature favors the nucleation and the crystal growth. The effect of Y^3+^ doping on the morphology and UC emission of the as-prepared samples were systematically investigated. The results demonstrate that Y^3+^ doping may cause the size-tuning and shape evolution of the crystals. Compared to their Y^3+^-free samples, the integrated spectral intensities in the range of 445–495 nm from α-, β-, and α/β-mixed NaLuF_4_:20%Yb^3+^, 1%Tm^3+^ crystals with 40 mol% Y^3+^ doping are increased by 9.7, 4.4, and 24.3 times, respectively; red UC luminescence intensities in the range of 630–725 nm are enhanced by 4.6, 2.4, and 24.9 times, respectively. It is proposed that the increased UC emission intensity is mainly ascribed to the deformation of crystal lattice, due to the electron cloud distortion in host lattice after Y^3+^ doping. Besides, as for Y^3+^ doped β-NaLuF_4_:20%Yb^3+^, 1%Tm^3+^ microcrystals, the larger-size (relative to Y^3+^-absent samples) of the samples with 40 mol% Y^3+^ doping may have a small contribution to the enhancement of UC luminescence intensity. As a result of their intense UC emission, these phosphors may be suitable for optoelectronic devices.

## Methods

### Chemicals

All of the chemicals are of analytical grade and used as received without further purification. 1 M of Lu(NO_3_)_3_, 1 M of Y(NO_3_)_3_, 0.5 M of Yb(NO_3_)_3_, and 0.1 M of Tm(NO_3_)_3_ stock solutions were prepared by dissolving the corresponding rare earth oxide (99.99%) in dilute nitric acid (30%) at elevated temperature.

### Preparation

All samples were prepared based on our previously reported procedures^22–24^. As for the synthesis of Y^3+^-absent α-NaLuF_4_:20%Yb^3+^, 1%Tm^3+^ nanocrystals, 3 mmol of citric acid (2 M, 1.5 mL), 5 mmol of NaOH (4 M, 1.25 mL) and 10 mL of deionized water were mixed and stirred for 10 min. Then 1 mmol of RE(NO_3_)_3_ (0.79 mmol of Lu(NO_3_)_3_ (1M, 0.79 mL), 0.2 mmol of Yb(NO_3_)_3_ (0.5 M, 0.4 mL), and 0.01 mmol of Tm(NO_3_)_3_ (0.1 M, 0.1 mL)) were added to above mixture and then stirred for 30 min to form the RE-Cit^3−^ complex. Subsequently, 16 mL of aqueous solution containing 9 mmol of NaF (1 M, 9 mL) and 7 mL of deionized water were added into the chelated RE-Cit^3−^ complex to form a colloidal suspension and kept stirring for another 30 min. Finally, the suspension was transferred into a 50 ml-Teflon vessel, sealed in autoclave and maintained at 110 °C for 12 h. After the autoclave was cooled to room temperature naturally, the final products separated by centrifugation, washed with ethanol and deionized water several times, and then dried in air at 60 °C for 12 h. Other samples were prepared by a similar process only by tuning the reaction temperature (110–200 °C) and Y^3+^ content (0–79 mol%).

### Characterization

The crystal structure of the as-prepared samples was confirmed by powder X-ray diffraction (XRD) patterns using the D-Max 2200VPC XRD from Rigaku Company (Cu-Kα radiation, λ = 1.5418 Å). The morphology was observed by Oxford Quanta 400 F Thermal Field Emission environmental Scanning Electronic Microscope (SEM). UC photoluminescence spectra were carried out on an Edinburgh Instrument Company FLS980 combined fluorescence lifetime and steady-state fluorescence spectrometer equipped with a 1 W 980 nm laser diode.
